# Activation of WIP1 Phosphatase by HTLV-1 Tax Mitigates the Cellular Response to DNA Damage

**DOI:** 10.1371/journal.pone.0055989

**Published:** 2013-02-06

**Authors:** Tajhal Dayaram, Francene J. Lemoine, Lawrence A. Donehower, Susan J. Marriott

**Affiliations:** 1 Interdepartmental Program in Cell and Molecular Biology, Baylor College of Medicine, Houston, Texas, United States of America; 2 Department of Molecular Virology and Microbiology, Baylor College of Medicine, Houston, Texas, United States of America; 3 Department of Biological Sciences, Northwestern State University of Louisiana, Natchitoches, Louisiana, United States of America; Yonsei University, Korea, Republic of

## Abstract

Genomic instability stemming from dysregulation of cell cycle checkpoints and DNA damage response (DDR) is a common feature of many cancers. The cancer adult T cell leukemia (ATL) can occur in individuals infected with human T cell leukemia virus type 1 (HTLV-1), and ATL cells contain extensive chromosomal abnormalities, suggesting that they have defects in the recognition or repair of DNA damage. Since Tax is the transforming protein encoded by HTLV-1, we asked whether Tax can affect cell cycle checkpoints and the DDR. Using a combination of flow cytometry and DNA repair assays we showed that Tax-expressing cells exit G_1_ phase and initiate DNA replication prematurely following damage. Reduced phosphorylation of H2AX (γH2AX) and RPA2, phosphoproteins that are essential to properly initiate the DDR, was also observed in Tax-expressing cells. To determine the cause of decreased DDR protein phosphorylation in Tax-expressing cells, we examined the cellular phosphatase, WIP1, which is known to dephosphorylate γH2AX. We found that Tax can interact with Wip1 *in vivo* and *in vitro*, and that Tax-expressing cells display elevated levels of Wip1 mRNA. I*n vitro* phosphatase assays showed that Tax can enhance Wip1 activity on a γH2AX peptide target by 2-fold. Thus, loss of γH2AX *in vivo* could be due, in part, to increased expression and activity of WIP1 in the presence of Tax. siRNA knockdown of WIP1 in Tax-expressing cells rescued γH2AX in response to damage, confirming the role of WIP1 in the DDR. These studies demonstrate that Tax can disengage the G_1_/S checkpoint by enhancing WIP1 activity, resulting in reduced DDR. Premature G_1_ exit of Tax-expressing cells in the presence of DNA lesions creates an environment that tolerates incorporation of random mutations into the host genome.

## Introduction

Cells have evolved biochemical pathways that detect DNA damage and arrest cell cycle progression to allow for DNA repair. For example, the G_1_/S checkpoint prevents cells from entering S-phase in the presence of DNA damage. Defects in this checkpoint can allow replication of damaged DNA and introduction of mutations into the genome. Molecular mechanisms that govern the proper induction and function of cell cycle checkpoints are disrupted in many forms of cancer [1]–[3], demonstrating their importance in maintaining proper cellular growth control. Cell cycle checkpoint dysregulation is also a recurring theme in virally associated cancers, emphasizing its key role in cellular transformation (reviewed in 4).

Upon sensing DNA damage, cells initiate a signaling cascade that stems from activation of the PI3K-like kinases ATM and ATR. These kinases phosphorylate a series of downstream effector proteins, including p53, to induce cell cycle arrest and DNA repair mechanisms. Following DNA repair, cells must recover from the checkpoint and resume normal cell cycle progression. Improper function of the G_1_/S phase checkpoint allows cells containing genomic lesions to progress into S phase and initiate DNA synthesis. Replication of DNA under these conditions could introduce a variety of genomic mutations, thus the DNA damage response (DDR) functions as an early barrier to tumorigenesis by preserving genomic integrity [4], [5].

Tax is a regulatory protein encoded by the transforming retrovirus human T cell leukemia virus type 1 (HTLV-1), the etiologic agent of the fatal human cancer, adult T cell leukemia (ATL) [6]. Tax is essential for HTLV-1 associated cellular transformation [7]–[9] and has been characterized as a viral oncoprotein [10]–[16]. In fact, Tax expression alone is sufficient to increase cellular mutation rates and have other deleterious effects on the host genome [17], [18].

ATL cells typically display extensive genome instability leading to chromosomal aberrations. Chromosomal defects, such as those seen in ATL cells typically result from defects in DNA damage induced cell cycle checkpoints. Proper execution of the G_1_/S phase DNA damage-induced cell cycle checkpoint induces cell cycle arrest and accumulation of cells in G_1_ phase of the cell cycle. This checkpoint is particularly important in preserving genomic integrity because cells that fail to properly arrest the cell cycle or repair damaged DNA enter S phase and replicate DNA in the presence of damage, thus allowing incorporation of mutations into the host genome.

Mechanisms governing checkpoint recovery are not as clearly understood as checkpoint activation. Since the DDR stems from activation of several kinases and phosphorylation of multiple proteins, one mode of checkpoint recovery involves activation or expression of phosphatases. In particular, the Wildtype p53-induced phosphatase 1 (WIP1) is emerging as a key player in the dephosphorylation and inactivation of p53 as well as several ATM/ATR target proteins (reviewed in 25). Thus, WIP1 can return cells to a prestressed state following proper DNA repair.

Since failure to establish a proper DDR can result in genomic instability due to ineffective repair of DNA lesions, we asked whether the DDR is properly executed in Tax expressing cells. In particular, we asked whether initiation of the DDR was affected by Tax and whether Tax-expressing cells were able to properly induce the G_1_/S cell cycle checkpoint to repair damaged DNA. Consistent with previously published work [19] we detected an abrogation of G_1_ cell cycle arrest following UV-damage. Our results further demonstrate that the checkpoint can be initiated but cannot be maintained. Since WIP1 may play an important role in G_1_/S checkpoint recovery, we analyzed the effects of Tax on WIP1 expression and function following UV-damage to determine whether WIP1 plays a role in premature checkpoint exit in Tax-expressing cells.

## Results

### Tax-expressing cells have a defect in G_1_ arrest following DNA damage

**Figure 1:**
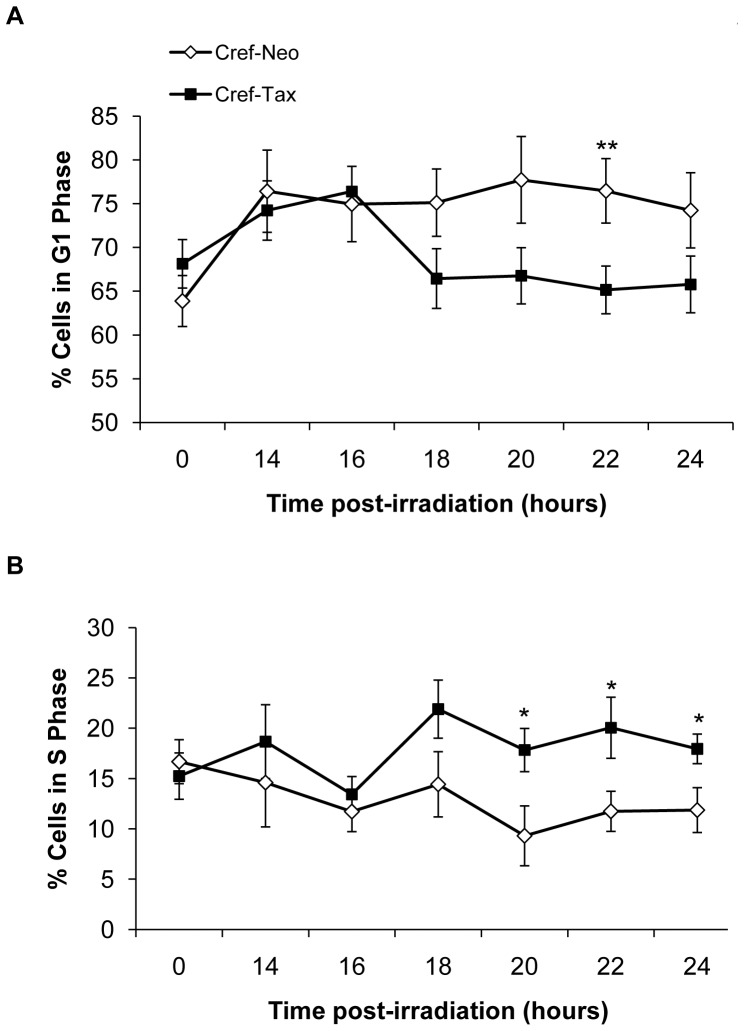
Tax expression alters the G_1_ phase arrest following DNA damage. Asynchronous CREF-neo and CREF-Tax cells were exposed to 30 J/m^2^ of UV irradiation. The percent of cells in G_1_ phase (A) and S phase (B) are displayed at the indicated times post-irradiation. Results shown are the average of three independent experiments (error bars represent standard error of the mean; * p-value≤0.1, ** p-value≤0.05).

Since proper induction of the G_1_/S phase DNA damage-induced cell cycle checkpoint results in cell cycle arrest and accumulation of cells in G_1_ phase, we first asked whether HTLV-I Tax affects the accumulation of cells in G_1_ phase of the cell cycle following UV-damage. Asynchronously growing CREF-neo and CREF-Tax cells were exposed to UV irradiation, and cell cycle progression was monitored. Consistent with proper induction of the G_1_/S phase DNA damage-induced cell cycle checkpoint, control CREF-neo cells arrested in G_1_ phase (Figure 1A), correlating with a reduction of cells in S (Figure 1B) and G_2_/M phases (data not shown) at 22 hours post-irradiation. In contrast, CREF-Tax cells displayed a transient increase in the percent of cells in G_1_ phase following UV damage, suggesting an initial arrest in G_1_ phase. However, after 16 hrs post-irradiation the percentage of Tax-expressing cells in G_1_ phase began to decline (Figure 1A) with a concurrent increase in cells in S phase (Figure 1B). These results suggest that Tax-expressing cells accumulate, at least briefly, in G_1_ phase following DNA damage (Figure 1A, 14 and 16 hr timepoints), then enter S phase earlier than control cells and led us to hypothesize that Tax expression disrupts the ability of cells to maintain a proper G_1_ arrest following UV-induced DNA damage.

**Figure 2:**
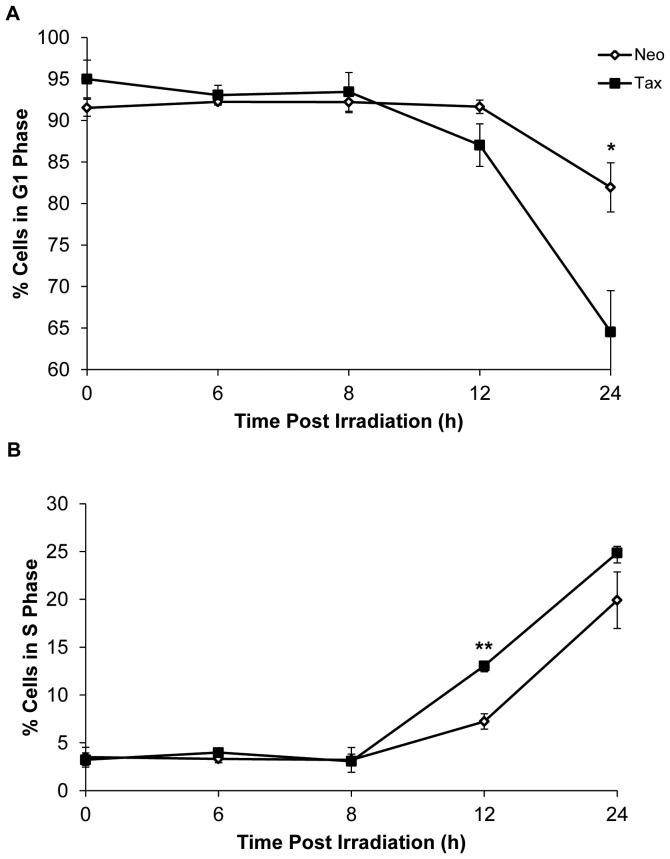
Tax expression accelerates S-phase entry following DNA damage. Synchronized CREF-neo and CREF-Tax cells were exposed to 30 J/m^2^ of UV irradiation 12 hours after release from G0, which is shown as “0” h post irradiation. The percent of cells in G1 phase (A) and S phase (B) are displayed at the indicated times post-irradiation. Results shown are the average of three independent experiments. (error bars represent standard error of the mean; * p-value≤0.1, ** p-value≤0.05).

To directly examine the effects of Tax on the G_1_/S DNA damage-induced cell cycle checkpoint, CREF-neo and CREF-Tax cells were synchronized in G_0_ by contact inhibition prior to UV-damage. This resulted in greater than 90% of the cells arresting in G_0_ and, upon splitting to a lower density, the cells were released into G_1_ [20]. We demonstrated previously that Tax expression affects neither the rate at which CREF cells synchronize nor the rate at which they enter the cell cycle following the release from such an arrest [20]. After G_0_ synchronization and subsequent release into G_1_ phase, CREF-neo and CREF-Tax cells were UV irradiated at 12 hours post-release, a point when both cell types were in G1, and their cell cycle progression was monitored by flow cytometry. Both CREF-Tax and CREF- neo cells show little to no change in the percentage of cells in G1 up to 8 hours post-UV suggesting that they are arrested in G1 phase (Figure 2A). CREF-Tax cells began to exit G1 phase between 8 and 12 hours post-UV irradiation, while CREF-neo cells exited G1 between 12 and 24 hours post-UV (Figure 2A). G1 exit coincided with an increased percentage of cells in S phase for each cell type (Figure 2B). Significantly more CREF-Tax cells than CREF-neo cells were in S phase at 12 hours post-UV damage (Figure 2B). These results demonstrated that Tax-expressing cells initiate a G1 arrest but fail to maintain the G_1_/S DNA damage-induced cell cycle checkpoint.

### Tax-expressing cells enter S phase in the presence of unrepaired DNA lesions

There are three possible mechanisms by which Tax-expression may accelerate entry into S phase following UV irradiation. First, Tax may accelerate the rate of DNA repair, such that when DNA damage occurs, the G_1_/S checkpoint is induced properly, the damage is quickly repaired, and the checkpoint is relieved faster than in control cells. Second, Tax-expressing cells may be less sensitive to DNA damage, in which case the G_1_/S checkpoint is induced, but there is less DNA damage to be repaired in cells expressing Tax, so repair can be completed in less time, and the checkpoint can be relieved earlier than in control cells. Lastly, cells expressing Tax may be able to overcome the G_1_/S checkpoint and enter S phase prior to completing DNA repair, doing so in the presence of unrepaired DNA damage.

**Figure 3:**
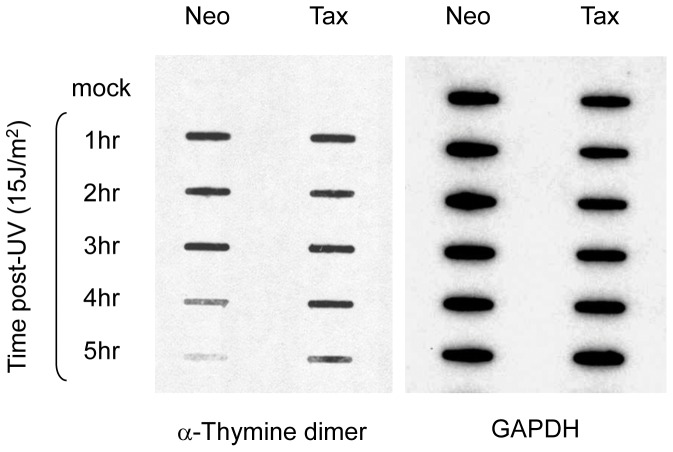
Tax represses repair of UV irradiation-induced DNA damage. Asynchronously growing CREF-neo and CREF-Tax cells were exposed to 15 J/m^2^ UV irradiation. Thymine dimers in genomic DNA were detected at indicated times following UV or mock-treatment.

To determine if Tax expression affected the rate of DNA repair or the level of DNA damage incurred by cells, we examined the presence of thymine-dimers over time in UV-damaged cells in the presence or absence of Tax. To ask whether Tax expression protects cells from incurring DNA damage, we examined the quantity of lesions induced by UV irradiation in CREF-neo and CREF-Tax cells by exposing them to UV irradiation and harvesting the DNA at different times post-UV. The genomic DNA isolated from these cells was blotted onto a membrane that was probed with an antibody specific for thymine dimers, the predominant DNA lesion caused by UV irradiation. By 1 hour post-UV treatment, equivalent levels of thymine dimers were observed in the presence or absence of Tax (Figure 3), indicating that Tax expression did not protect cells from DNA damage. However, at later timepoints (4–5 hrs post-UV) thymine dimers were more abundant in CREF-Tax cells than in CREF-Neo cells at the same timepoints (Figure 3), suggesting that Tax-expressing cells have a defect in the repair of these lesions. These results are consistent with previous work demonstrating that Tax can repress the repair of UV-damaged DNA [21]. Thus, the ability of Tax-expressing cells to enter prematurely into S phase following UV irradiation cannot be attributed to accelerated DNA repair. These results, together with the finding that Tax-expressing cells can enter S phase prematurely, support the hypothesis that Tax-expressing cells overcome the G_1_/S checkpoint and enter S phase in the presence of unrepaired DNA damage.

### Tax-expressing cells have diminished γH2AX and p-RPA foci following UV-damage

**Figure 4:**
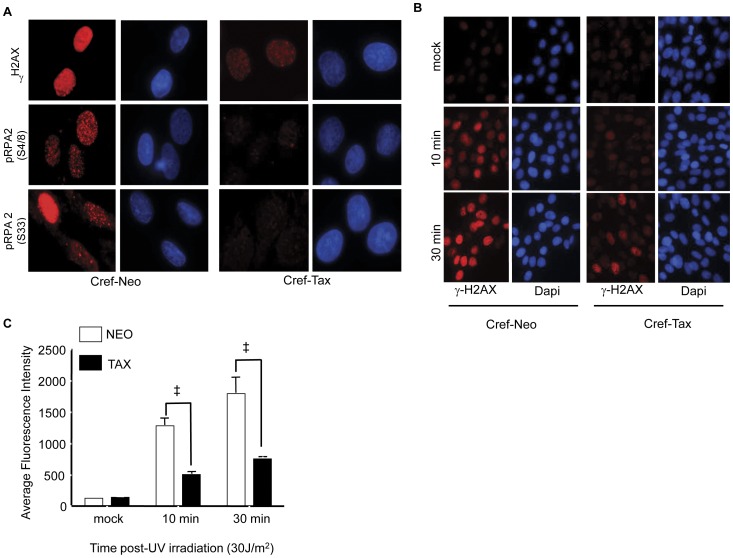
Tax expressing cells fail to form UV-induced γH2AX and p-RPA foci. (**A**) UV irradiated CREF-neo and CREF-Tax cells were stained with either anti- p-RPA2(S33), anti- p-RPA2(S4/8), or anti-γH2AX (all stained in red). Cells were counterstained with Dapi (blue) to visualize nuclei. (**B**) CREF-Neo and CREF-Tax cells were either mock-irradiated or damaged with 30 J/m^2^ UV. Cells were collected at the indicated timepoints and stained for γH2AX (red) and DAPI (blue). (**C**) Quantitation of γH2AX immunofluorescence intensity of 12 fields of at least 20 cells. Error bars represent standard error and ‡ represents a p-value≤0.001.

The initial accumulation of CREF-Tax cells in G_1_ following UV-damage (Figure 1) suggests that a G_1_/S checkpoint may be transiently activated. We therefore examined the effects of Tax on initiation of the DDR. One of the earliest effects of DNA damage is phosphorylation of histone H2AX and replication protein A (RPA2) that allows for the recruitment of ATM and ATR to sites of DNA damage. ATM and ATR can then subsequently phosphorylate multiple effector proteins to activate checkpoints. To determine whether Tax expression affects the initiation of the DDR we analyzed the phosphorylation of H2AX (γH2AX) and RPA (p-RPA2) in CREF-neo and CREF-Tax cells following UV-irradiation. RPA can be phosphorylated on multiple sites following DNA damage. We chose to analyze the effects of Tax on the phosphorylation of serines 4, 8 and 33, which are consensus sequences targeted by ATM/ATR (reviewed in [22]). Cells were exposed to UV irradiation and allowed to recover for 4 hours, at which point they should still be arrested in G_1_ (see Figure 1A). CREF-neo cells had detectable levels of γH2AX, p-RPA2 (S33) and p-RPA2 (S4/S8) while CREF-Tax cells had greatly reduced levels of these phosphoproteins (Figure 4A). While these phosphoproteins were diminished in CREF-Tax cells, they were not completely absent suggesting that the DDR can be initiated but not amplified in these cells.

We therefore examined γH2AX foci formation immediately following UV irradiation. CREF-neo and CREF-Tax cells were either mock-treated or exposed to UV-irradiation. Cells were collected at 10 and 30 minutes post-UV irradiation and analyzed for γH2AX by immunofluorescence. As early as 10 minutes following UV irradiation γH2AX levels were higher in both CREF-neo and CREF-Tax cells than in matched mock treated cells (Figure 4B). Although CREF-Tax cells had higher γH2AX levels at 10 and 30 minutes post-damage than did mock treated cells, they contained less γH2AX than CREF-Neo cells at the same timepoints (Figures 4B and 4C). The increase in γH2AX in CREF-Tax cells following UV-irradiation supports the idea that the DDR can be initiated in these cells, but that Tax prevents the accumulation of γH2AX. The initial accumulation of γH2AX could initiate a G_1_/S checkpoint however, the lower levels and inability to amplify γH2AX in Tax-expressing cells may allow early cell cycle exit.

**Figure 5:**
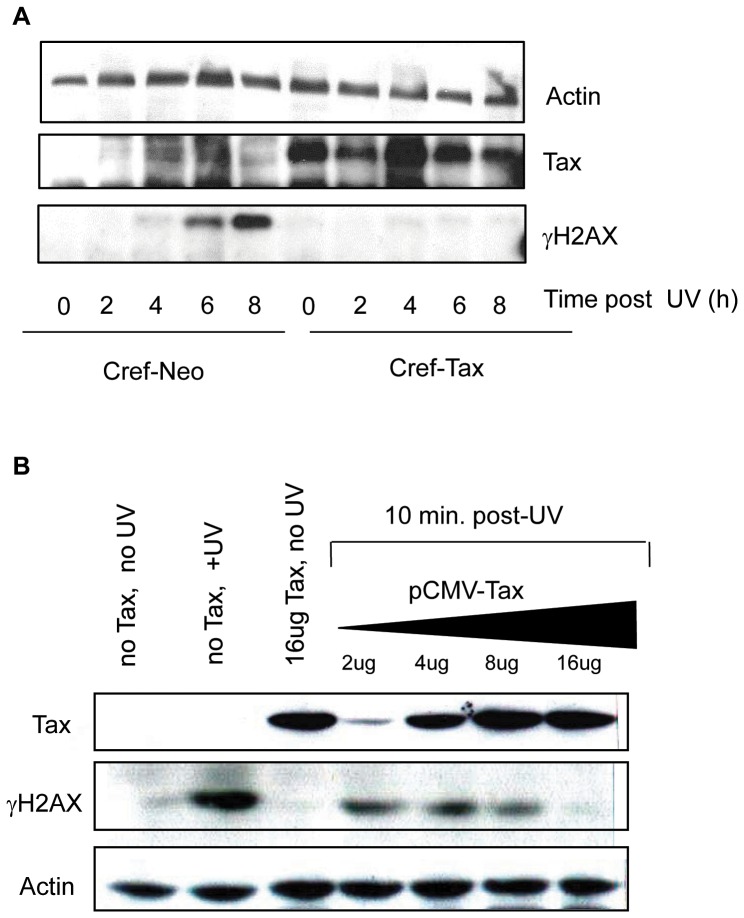
Tax expression inhits γH2AX in a dose-dependent manner. (**A**) CREF-neo and CREF-Tax cells were exposed to 30 J/m^2^ UV and harvested at the indicated timepoints. Whole cell extracts were analyzed by western blot for Actin, Tax and γH2AX. (**B**) 293 cells were untransfected (No Tax) or transfected with the indicated amounts of a Tax expression vector and exposed to 30 J/m^2^ UV. Cells were harvested at 10 minutes post-UV and whole cell extracts were analyzed by western blot for Tax, Actin and γH2AX.

Since it took 8 hours for CREF-Tax cells to begin to exit G1 following damage (Figure 2A and B) we wanted to ensure that γH2AX levels did indeed remain low in CREF-Tax cells prior to that timepoint post-damage. We, therefore, analyzed γH2AX levels by western blot from 0 to 8 hours post-UV damage. While γH2AX accumulation can be seen in CREF-neo cells from 4 to 8 hours post-UV, there is little to no detectable γH2AX in CREF-Tax cells by western blot (Figure 5A). This supports the results shown in Figure 4 and suggests that Tax-expressing cells are unable to maintain γH2AX expression following DNA damage. To further demonstrate that these results are specific to Tax expression and not due to effects that may arise from stable expression of Tax, we analyzed the effects of transiently expressed Tax on γH2AX at an early timepoint post-UV damage. 293 cells were transfected with increasing amounts of Tax and exposed to UV-damage prior to analysis by western blot. In the absence of Tax, γH2AX is easily detected in these cells post-damage, however with increasing levels of Tax expression we see decreasing levels of γH2AX (Figure 5B). These results support the fact that Tax-expressing cells have a defect in γH2AX accumulation following UV-damage.

### Tax expression enhances WIP1 expression at earlier times post-UV

The reduced levels of γH2AX in Tax-expressing cells following DNA damage could be due to a defect in H2AX phosphorylation or to increased dephosphorylation of γH2AX. Indeed, γH2AX dephosphorylation may play a critical role in G_1_/S checkpoint recovery. Thus, we next wanted to investigate whether the reduced levels of γH2AX in Tax-expressing cells result from the activation of a cellular phosphatase. Although several phosphatases have been implicated in γH2AX dephosphorylation, we analyzed the effect of Tax on the damage-induced phosphatase WIP1 that has been shown to dephosphorylate ATM, γH2AX, Chk1 and Chk2 [23]–[26]. Since WIP1 is a damage-induced phosphatase that targets multiple proteins following a variety of cellular stresses, its inhibition is consistent with the effects of Tax following DNA damage. WIP1 expression is low in unstressed cells. However, in response to DNA damage WIP1 mRNA is upregulated in a p53-dependent manner [27].

**Figure 6:**
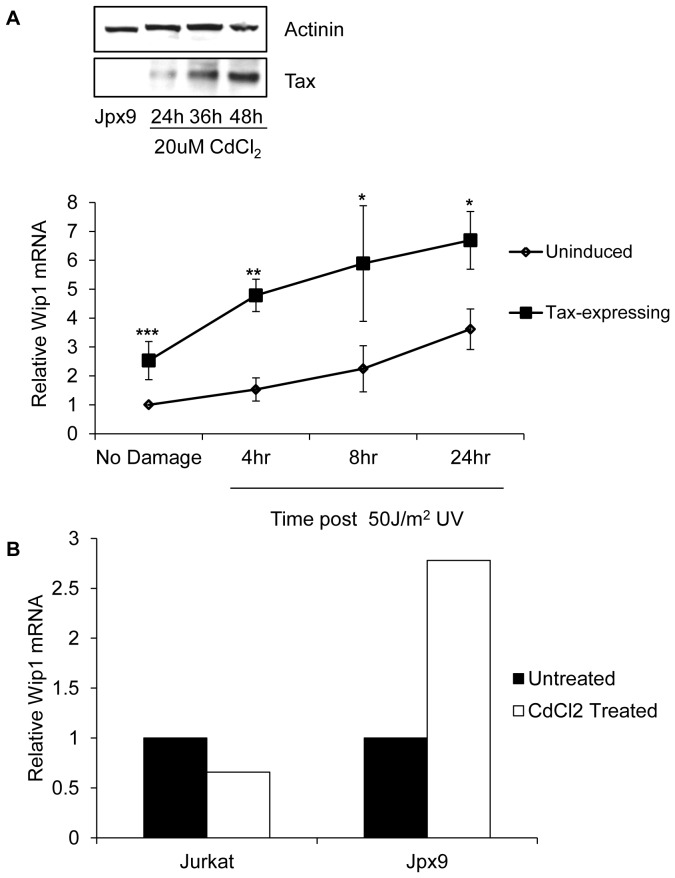
Tax-expressing cells upregulate WIP1 mRNA following UV-damage. (**A**) Jpx9 cells were induced for Tax expression with 20 uM CdCl_2_ and harvested at the indicated timepoints to assay for Tax expression by western blot. Uninduced or Jpx9 cells induced for 48 hours when Tax expression was strong were undamaged or exposed to 50 J/m^2^ UV and harvested at the indicated times for quantitative RT-PCR analysis. The y-axis represents WIP1 mRNA levels normalized to GAPDH. Relative WIP1 mRNA is shown in comparison to undamaged, uninduced Jpx9 cells. The average of three independent experiments is shown. Error bars represent standard error and asterisks indicate significant differences between Tax-expressing and uninduced cells at each timepoint (* = p≤0.1, ** = p≤0.05 and ***≤0.01). (**B**) Jurkat and Jpx9 cells were left untreated or treated with 20 µM CdCl_2_ for 48 hours. Cells were then harvested and resulting RNA subjected to quantitative RT-PCR for WIP1 and GAPDH. Relative WIP1 mRNA of treated cells is shows in comparison to untreated cells.

We used a Tax-inducible T-cell line (Jpx9) to analyze the effects of Tax-expression on WIP1 mRNA in response to UV irradiation. Jpx9 cells were induced with CdCl_2_ for 48 hours to induce Tax expression prior to UV-damage (Figure 6A inset). Uninduced and induced Jpx-9 cells were exposed to UV-irradiation and collected at various timepoints. The presence of WIP1 mRNA was analyzed in these samples using quantitative RT-PCR. Undamaged Tax expressing cells had twice as much WIP1 mRNA as undamaged cells without Tax expression (Figure 6A), which might reflect Tax activation of the WIP1 promoter. At 4 hours post-irradiation, Tax-expressing cells showed increased levels of WIP1 mRNA, with approximately 4-fold more WIP1 mRNA than in uninduced cells. Uninduced cells, however, did not show a significant increase in WIP1 mRNA levels until 24 hours post-irradiation. WIP1 mRNA levels increased in both Tax-expressing and uninduced cells after UV-damage, however, Tax-expressing cells consistently had higher levels of WIP1 mRNA. To ensure that the increased WIP1 mRNA seen in induced Jpx9 cells was due to Tax expression and not simply a result of CdCl_2_ treatment, we examined the effects of CdCl_2_ treatment in the parental Jurkat cell line. Jurkat and Jpx9 cells were treated with CdCl_2_ and WIP1 mRNA was analyzed by quantitative RT-PCR. While CdCl_2_ treatment in Jpx9 cells resulted in increased levels of WIP1 mRNA, CdCl_2_ did not affect WIP1 mRNA levels in Jurkat cells (Figure 6B). Therefore, the upregulation of WIP1 in CdCl_2_ induced Jpx9 cells following DNA damage could be attributed to Tax expression.

### Tax interacts with the damage-induced phosphatase WIP1 and enhances WIP1 phosphatase activity

**Figure 7:**
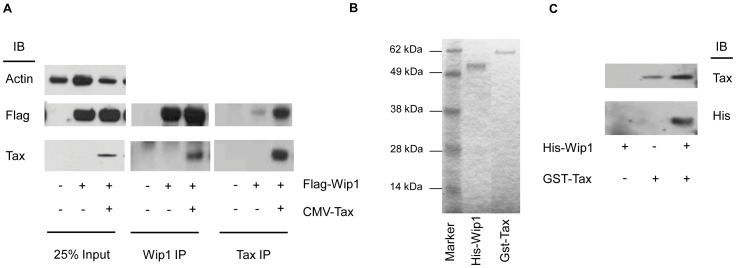
Tax interacts with WIP1. (**A**) 293 cells were transfected with either a Flag-WIP1 construct alone or Flag-WIP1 along with a Tax expression construct. Lysates were immunoprecipitated using either anti-WIP1 or anti-Tax antibodies followed by western blotting with anti-Flag or anti-Tax antibodies. (**B**) Coomassie stain of purified His-WIP1 and GST-Tax used in panel C. (**C**) His-WIP1 was incubated with either Glutathione sepharose beads or GST-Tax immobilized on Glutathione sepharose. Reactions were analyzed by western blot with anti-Tax and anti-His antibodies.

Since Tax is known to interact with a variety of cellular proteins, including another cellular phosphatase, PP2A [28], we asked whether Tax could interact with WIP1. Flag-WIP1 and Tax expression plasmids were co-transfected into 293 cells and co-immunoprecipitation (co-IP) was used to determine whether Tax and WIP1 interact *in vivo*. Tax was detected in western blots of co-transfected cellular lysates immunoprecipitated using an anti-WIP1 antibody, but not in lysates from control cells transfected with Flag-WIP1 only (Figure 7A). Reciprocal co-IPs using an anti-Tax antibody confirmed the *in vivo* interaction between Tax and Flag-WIP1. To determine whether Tax and WIP1 interact *in vitro*, purified GST-Tax was immobilized on glutathione-sepharose beads, incubated with His-WIP1 (Figure 7B), and proteins retained on the beads were analyzed by western blot using an anti-His antibody. His-WIP1 was recovered from beads, incubated with GST-Tax but not from beads alone (Figure 7C). Together, these results indicate that Tax can interact with WIP1 both *in vivo* and *in vitro*.

**Figure 8:**
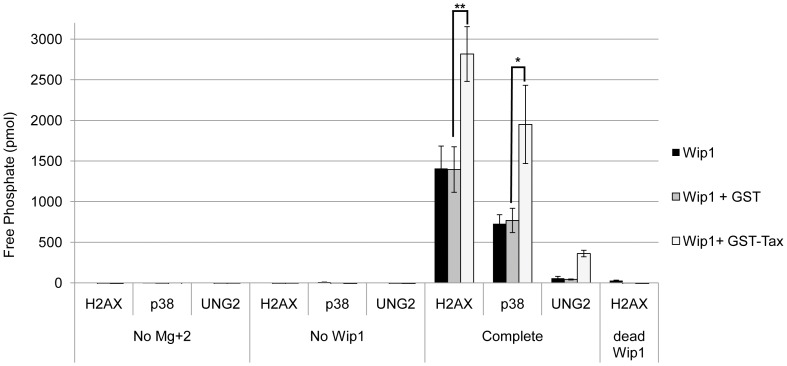
Tax enhances WIP1 phosphatase activity. *In vitro* phosphatase assays were performed using purified His-WIP1, GST or GST-Tax, and H2AX (pS139), p38 (pT180) and UNG2 (pT31) phosphopeptides. Free phosphate levels for each reaction are shown. The average of three independent experiments is shown. Error bars represent standard error and asterisks indicate significance (* = p≤0.1 and ** = p≤0.05).

To determine whether the interaction of Tax with WIP1 could affect WIP1 phosphatase activity, we used H2AX (pS139) and p38 MAP kinase (pT180) phosphopeptides in an *in vitro* phosphatase assay. Both of these phosphopeptides are efficiently dephosphorylated by recombinant Wip1 in vitro [26], [29]. Incubation of the H2AX phosphopeptide with purified His-WIP1 alone or His-WIP1 with purified GST protein released similar levels of free phosphate indicating comparable levels of WIP1 phosphatase activity (Figure 8). Incubation of His-WIP1 with GST-Tax produced approximately 2-fold more WIP1 activity on H2AX phosphopeptide substrates, suggesting that Tax can enhance WIP1 phosphatase activity. Incubation of GST-Tax with the H2AX phosphopeptide in the absence of His-WIP1 did not release any free phosphate, indicating that dephosphorylation of the H2AX phosphopeptide by Tax required WIP1 and is not a result of direct phosphatase activity of Tax. A His-WIP1 protein harboring a point mutation that renders the protein enzymatically inactive (dead WIP1) was also tested to ensure that the effects of Tax on H2AX was mediated through WIP1. As expected, no phosphate was released when this phosphatase dead WIP1 was incubated with or without Tax. The specificity of enhanced His-WIP1 dephosphorylation by Tax was determined using an UNG2 phosphopeptide, which contains a phosphorylation site that is not targeted by WIP1. As expected, there was minimal release of free phosphate from the UNG2 phosphopeptide in the presence of His-WIP1 only, His-WIP1 with GST, or His-WIP1 with GST-Tax (Figure 8).

To determine whether the effect of Tax on WIP1 was specific for ATM/ATR target proteins or whether Tax affected overall WIP1 activity, we used a phosphopeptide containing a known WIP1 target site on p38 that is not responsive to ATM/ATR. Similar to the results seen with γH2AX phosphopeptide, incubation of His-WIP1 with GST-Tax released approximately 2-fold more free phosphate from the p38 phosphopeptide than His-WIP1 alone (Figure 8). Overall, these results indicate that the interaction of Tax with WIP1 enhances WIP1 phosphatase activity, which is consistent with the premature dephosphorylation of γH2AX in Tax-expressing cells. The significant increase in WIP1 mRNA in Tax-expressing cells at the earliest timepoint post-UV (Figure 6A) together with increased WIP1 phosphatase activity (Figure 8) could prematurely dephosphorylate γH2AX early in the DNA damage response.

### Loss of WIP1 in Tax-expressing cells enhances γH2AX after DNA damage

**Figure 9:**
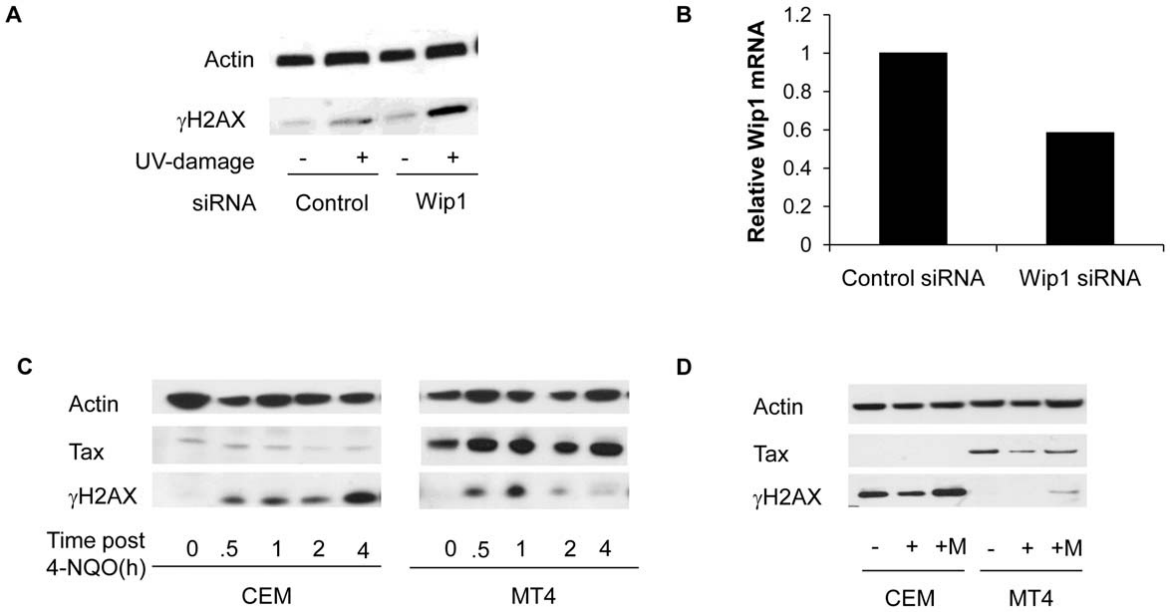
Inhibition of WIP1 in Tax-expressing cells restores γH2AX levels following DNA damage. (**A**) CREF-Tax cells were transfected with a control siRNA or siRNA targeted to WIP1. 48 hours post-transfection cells were treated with 30 J/m^2^ UV, allowed to recover for 4 hours, and analyzed by western blot for γH2AX and actin. (**B**) UV-damaged control siRNA transfected and WIP1 siRNA transfected CREF-Tax cells were analyzed by quantitative RT-PCR for WIP1 expression. (**C**) Uninfected (CEM) and HTLV-1 infected (MT4) cells (untreated or treated with the UV-mimetic drug 4-NQO) were harvested at the indicated times and analyzed by western blot. (**D**) CEM and MT4 cells were left untreated (−), treated with 4-NQO (+), or treated with the WIP1 inhibitor Compound M for 1 hour followed by 4-NQO (+M) and harvested after a 4 hour recovery followed by analysis by western blot for γH2AX.

Since WIP1 appears to play a role in the dephosphorylation of γH2AX after DNA damage in Tax-expressing cells, we asked whether loss of WIP1 in these cells would enhance γH2AX. CREF-Tax cells were transfected with an siRNA targeted to WIP1or a control scrambled siRNA. Cells were then either left undamaged or exposed to UV-irradiation and allowed to recover for 4 hours, a timepoint at which low levels of γH2AX are detected by IF (Figure 4) and western blot (data not shown). Quantitative RT-PCR of WIP1 mRNA in UV-damaged cells showed a 40% reduction in WIP1 levels following WIP1 siRNA treatment (Figure 9B). Both control siRNA and WIP1 siRNA transfected cells showed minimal levels of γH2AX in undamaged cells. Control siRNA cells exposed to UV irradiation showed a small increase in γH2AX, however WIP1 siRNA treated cells showed significantly higher levels of γH2AX than did control cells (Figure 9A). These results demonstrate that loss of WIP1 in CREF-Tax cells restores the accumulation of γH2AX following DNA damage, supporting a role for WIP1 in Tax-mediated attenuation of DDR.

To examine the effects of Tax and WIP1 in T-cells, the natural target of HTLV-1, uninfected (CEM) and HTLV-1 infected (MT4) cells were exposed to the UV-mimetic drug 4-NQO and harvested at various timepoints to analyze H2AX phosphorylation. In CEM cells, which do not express Tax, γH2AX was detectable as early as 30 minutes after 4-NQO treatment and continued to increase through 4 hours post-irradiation (Figure 9C). Tax-expressing MT4 cells also show enhanced γH2AX levels at early timepoints post 4-NQO treatment as compared to undamaged cells, however, these γH2AX levels decreased by 2 and 4 hours following 4-NQO treatment (Figure 9C). This result indicates that the γH2AX defect in this HTLV-1 infected cell line is similar to that observed in CREF-Tax cells.

To determine whether γH2AX levels can be restored following DNA damage we inhibited WIP1 activity in CEM and MT4 cells using a chemical inhibitor (Compound M) identified in a screen for WIP1 suppressors [30]. Compound M has been shown to inhibit WIP1 activity, but not the PP2C or PP2A phosphatases [30]. Compound M was added to cells 1 hour prior to 4-NQO treatment and cells were collected 4 hours later for western blot analysis. γH2AX levels in 4-NQO treated MT4 cells were modestly higher than untreated cells (Figure 9D). Treatment with Compound M prior to damage resulted in significantly more γH2AX than in damaged cells in the absence of Compound M (Figure 9D). This result supports our finding that inhibition of WIP1 in CREF-Tax cells enhances γH2AX following DNA damage.

## Discussion

The results presented here demonstrated that Tax expression alters the ability of cells containing UV-induced DNA damage to arrest in G_1_ phase. This is consistent with previous results showing an attenuated G_1_ checkpoint following UV damage [19]. Although these authors suggested that Tax-expressing cells fail to arrest in G_1_ phase, we showed that following UV irradiation, cells expressing Tax exhibited a transient G_1_ phase arrest prior to premature S phase entry. The exact mechanism by which Tax-expressing cells induce a p53-independent arrest is unclear, but p53-deficient human tumor cell lines, as well as p53^−/−^ mouse skin fibroblasts, are capable of arresting in G1 following UV-damage [31].

What are the consequences of entering S phase in the presence of DNA damage? It has been demonstrated previously in cells undergoing DNA replication in the presence of damage that lesions, such as those resulting from UV irradiation, serve as blocks to progressing DNA replication forks. The resolution of such stalled replication forks is not well understood, but has been shown to result in an overall slowing of the DNA replication process. In fact, induction of DNA damage in cells deficient in DNA repair pathways, such as NER, results in slower DNA replication and an elongated S phase [32], an effect likely due to the longer time required to resolve stalled or blocked replication forks. Since Tax has been shown to repress the repair of DNA damage (Figure 3) yet allow entry into S phase following UV irradiation (Figure 1), the elongated S phase observed in Tax-expressing cells is consistent with the presence of unrepaired replication blocking lesions that slow the process of DNA replication and initiate an intra-S checkpoint.

The effects of Tax on “normal” cell cycle progression have been characterized previously. In addition to its ability to stimulate cell cycle entry from quiescence [33], [34], Tax expression has been shown to induce an accelerated G_1_ phase progression resulting in a shorter time required to complete cell division [35]. Although the exact molecular mechanism by which Tax stimulates cell cycle progression has not been elucidated, the ability of Tax to inhibit the function of negative cell cycle regulators such as p16 [33], [33], [36,37], p53 [38]–[40], and pRb [41]–[43] and to activate positive cell cycle regulators such as E2F [41], [43], [44], cdk4 [34], [43], [45], cdk6 [34], and cdk2 [20] likely is involved in this effect. Through these activities, Tax expression confers a specific growth advantage to cells. This growth advantage, in conjunction with the ability of Tax-expressing cells to undergo DNA replication in the presence of damage as described in this report, may establish an environment in which a variety of random mutations are introduced into the genome, and some of these mutations may provide additional growth advantages. Over time, a specific combination of random genomic mutations may allow a subset of HTLV-1 infected cells to escape normal cellular growth control and become transformed. It is likely that the ability of Tax to stimulate cell cycle progression under both normal and abnormal (i.e. in the presence of DNA damage) conditions plays an important role in establishing Tax-mediated, HTLV-1 associated cellular transformation.

The presence of decreased γH2AX in Tax-expressing cells following UV-irradiation is similar to previous results from our laboratory demonstrating decreased γH2AX in response to double strand breaks in CREF-Tax cells [46]. Our previous work showed robust γH2AX and phosphorylated ATM (p-ATM) immediately following double strand breaks in Tax-expressing and non-Tax expressing cells [46]. However, γH2AX levels were prematurely attenuated in the presence of Tax as compared to control cells. Loss of γH2AX in the presence of Tax could account for the lack of p-ATM in these cells and therefore premature exit from the damage induced checkpoint. A defect in the accumulation of γH2AX in Tax-expressing cells exposed to ionizing irradiation was also reported by Durkin et al. [47], suggesting that this may be a general response of Tax-expressing cells to multiple types of damage. Diminished γH2AX in response to both UV-induced DNA damage and double strand DNA breaks is consistent with an initial phosphorylation of H2AX in the presence of Tax, but an inhibition of γH2AX accumulation, which introduces a major barrier to checkpoint maintenance. Here we've shown that loss of WIP1 in Tax-expressing cells can rescue γH2AX levels following UV-damage. This suggests that in Tax-expressing cells WIP1 could play a role in premature checkpoint exit as marked by loss of γH2AX and p-ATM in the case of double strand breaks.

An *in vivo* role for WIP1 in p53 inactivation in Tax-expressing cells was recently reported by Zane et al. [48]. Loss of WIP1 in Tax transgenic mice resulted in longer tumor-free survival [48], suggesting that the effects of WIP1 on cell cycle checkpoints in Tax-expressing cells could involve effects on p53. Our findings differ somewhat from those of Zane et al. as these authors found no difference between WIP1 mRNA levels in HTLV-1 infected T-cell lines and uninfected T-cell lines, while we saw slightly higher levels of WIP1 mRNA in Tax-expressing Jpx9 T-cells. The difference between our results and those of Zane could be due to differences in the cell lines used. We also saw a significant increase in WIP1 mRNA levels following DNA damage in Tax-expressing cells, which was not examined by Zane et al.

We attribute the decreased phosphorylation of H2AX in Tax-expressing cells to enhanced activity and expression of WIP1 following UV irradiation. Tax appears to have a dual effect on WIP1, affecting not only WIP1 expression but also WIP1 phosphatase activity. The mechanism by which Tax affects WIP1 expression is unclear since WIP1 mRNA expression is p53-dependent following DNA damage and Tax is known to functionally inhibit p53. Because Tax is known to affect the transcription of multiple cellular genes in an NF-κB and CREB dependent manner [49]–[56], CREB and NF- κB responsive elements in the WIP1 promoter [27], [57]–[59] may allow Tax activation of WIP1 transcription.

It is intriguing that Tax binding to WIP1 appears to stimulate WIP1 phosphatase activity. The 3-dimensional structure of WIP1 is currently unknown and the exact mechanism of WIP1 phosphatase activity is yet to be elucidated. However, modeling of WIP1 based on PP2Cα, a member of the same phosphatase family, suggests that specific amino acids may be important for WIP1 catalytic activity based on their binding to the phosphate of target substrates [60]. The interaction domains of WIP1 and Tax have not been mapped but we speculate that Tax binds to WIP1 inducing a conformational change that enhances its catalytic activity or provides greater access to the target phosphorylation sites of the substrate.

The increase in WIP1 mRNA levels in undamaged Tax-expressing cells could account for the overall diminished levels of γH2AX at early timepoints post-damage in these cells. Inhibition of γH2AX accumulation early in the DDR could dampen the overall level of damage response and allow for quicker checkpoint recovery in the presence of Tax. The effects of Tax on WIP1 appear to attenuate the DNA damage response by prematurely dephosphorylating γH2AX, thus releasing cells from the cell cycle checkpoint and allowing S-phase entry prior to the completion DNA repair. This is consistent with the fact that WIP1 has been shown to dephosphorylate a number of targets in the ATM/ATR-mediated DDR as well as suppress the critical G1 checkpoint regulator p53 [61]. The ability of Tax to dysregulate the G_1_/S phase DNA damage-induced cell cycle checkpoint establishes an environment conducive for the formation of DNA breaks and mutations leading to chromosomal aberrations that are characteristic of HTLV-I associated transformation and the development of ATL.

## Materials and Methods

### Cell Lines

CREF-neo and CREF-Tax cells are previously-described clonal rat embryo fibroblasts (CREFs) that stably express a neomycin or Tax expression construct respectively [62]. Briefly, CREF cells were transfected with Tax and neomycin resistance plasmids and selected for resistant clones in G418. These cells were maintained in media containing 600 µg/ml geneticin (Invitrogen, Carlsbad,CA.) to select cells that maintain the plasmids. Resistant clones were then pooled. Tax expression in the CREF-Tax cells is similar to that seen in the MT4 HTLV-1 infected T-cell line. CREF-neo, CREF-Tax and 293 cells were maintained in Dulbecco's modified Eagle's media supplemented with 10% fetal bovine serum. Jpx-9 T lymphocytes were derived from Jurkat cells by stable transfection of a Tax expression plasmid under control of the metallothionine promoter [63]. Jpx-9 cells were maintained in RPMI media containing 10% FBS and supplemented with 600 µg/ml geneticin. Where indicated, CdCl_2_ was added to the culture media at a final concentration of 20 µM for 48 hours prior to UV-treatment to induce Tax expression. CEM and MT4 cells (NIH AIDS Reagents Program) were maintained in RPMI media containing 10% FBS. All cells were grown in a humidified atmosphere at 37°C in 5% CO_2_.

### Antibodies

Anti-cdk2, anti-actinin and anti-His antibodies were purchased from Santa Cruz Biotechnology (Santa Cruz, CA). Anti-actin and anti-Flag antibodies were purchased from Sigma (St. Louis, MO). When indicated, actinin was used in place of actin as a loading control to allow separation of specific molecular weights. The anti-thymine dimer antibody (Clone KTM53) was purchased from Kamiya Biomedical Company (Seattle, WA). Anti-Tax monoclonal antibody Tab170 was obtained from the AIDS Research and Reference Reagent Program, (Germantown, MD) and rabbit anti-Tax serum 586 was obtained from John Brady (NCI, National Institutes of Health). Anti-γH2AX (clone JBW301) was purchased from Millipore (Billerca, MA). Anti-WIP1, anti-pRPA2 (S33) and anti-pRPA2 (S4/S8) were purchased from Bethyl Laboratories (Montgomery, TX).

### UV Irradiation

For UV irradiation, media was removed and cells were washed once with PBS. A Stratlinker (Stratagene, La Jolla, CA.) was used to deliver the indicated dose of UV-C irradiation to the plates. Original media was then returned and cells recovered for the indicated times.

### Propidium Iodide Staining and Flow Cytometry

Cells were collected, and 1×10^6^ cells were resuspended in 2 mL of 0.9% NaCl and fixed with 5 mL of 95% ethanol. Fixed cells were incubated at room temperature for at least 30 minutes and stored at 4°C. To stain, the cell pellet was resuspended in 0.5 mL of 50 µg/mL propidium iodide (PI) (Sigma, St. Louis, MO) and 0.1 mL of 1 mg/mL RNase A (Sigma, St. Louis, MO) and incubated at 37°C for 30 minutes. A flow cytometer (Epic Profile, Coulter, CO) was used to analyze the cell cycle distribution. The percentage of cells in each phase of the cell cycle was determined using ModFit (Verity).

### Cell Synchronization

The G_0_ synchronization of cells was performed as previously described [[20]. In short, CREF cells were seeded into 100-mm dishes at a density of 5×10^4^ cells/mL. The cells were allowed to reach confluence and were maintained at 100% confluence for 48 hours. To release from the synchronized arrest, cells were split 1∶12 into new 100-mm dishes with fresh media.

### Host Cell Reactivation Assay

The host cell reactivation (HCR) assay was performed as previously described [21]. In short, a pMSV-Luc reporter plasmid was irradiated with 1000 J/m^2^ UV-C then co-transfected by calcium phosphate precipitation into CREF cells along with a Tax expression (pSV_2_-Tax) or control expression (pSV_2_-neo) plasmid and an undamaged CAT reporter plasmid. Forty-eight hours later, cells were harvested and luciferase activity was analyzed using a Turner TD-20e luminometer. Transfection efficiency, as measured by CAT activity, was used to normalize luciferase activity for each sample. The level of luciferase activity in extracts from CREF cells transfected with a mock-treated luciferase reporter was set at 100%. Repair activity of cells transfected with a UV irradiated luciferase reporter and either a neomycin or Tax expression plasmid, is reported as a percentage of the activity seen with the mock-irradiated reporter.

### Genomic DNA Isolation

Cells were collected, resuspended in lysis buffer (50 mM Tris pH 7.5, 50 mM EDTA, 100 mM NaCl, 1% SDS, 5 mM DTT) and 100 µg of Proteinase K, and incubated at 65°C overnight. Samples were subjected to phenol∶chloroform extraction followed by ethanol precipitation. DNA then was resuspended in TE (10 mM Tris-HCl pH 8.0, 1 mM EDTA) and quantitated using a spectrophotometer.

### Analysis of Thymine Dimers

DNA was denatured by boiling for 10 minutes followed by chilling quickly on ice. Ammonium acetate was added to stabilize the denatured DNA, and equivalent amounts of each sample were blotted onto positively charged nylon membranes (Boehringer Mannheim) using a Slot Blot Minifold II (Schleicher & Schuell, Keene, NH). The membrane was fixed at 120°C for 30 minutes, blocked for 1 hour in PBS containing 5% non-fat dried milk then incubated with an HRP-conjugated anti-thymine dimer antibody overnight at 4°C. Membranes were subsequently washed prior to Southern blot analysis using a P^32^ labeled GAPDH probe (Sigma, St. Louis, MO) as a DNA loading control.

### Immunoprecipitations

293 cells were transfected with Flag-WIP1 [64] alone or together with a CMV-Tax plasmid that has previously been described [65]. Nuclear lysates and immunoprecipitations were performed using the Active Motif Nuclear Complex Co-IP Kit according to the manufacturer's instructions (Active Motif, Carlsbad CA).

### Immunofluorescence

Cells were seeded onto coverslips and allowed to grow to 50% confluency prior to UV treatment. Cells were subsequently fixed in ice-cold methanol for 10 minutes then washed three times in PBS-T (PBS +0.1% TWEEN-20). Incubation with primary antibodies diluted in 5% BSA/PBS-T was performed overnight at 4°C. Cells were washed three times in PBS-T prior to incubation with fluorophore-conjugated secondary antibody (Invitrogen, Carlsbad, CA) diluted in PBS-T for 30 minutes at room temperature. Cells were then counterstained with DAPI (4′,6′-diamidino-2-phenylinodole) (Sigma, St. Louis, MO) to visualize nuclei and mounted using ProLong Gold Antifade (Invitrogen, Carlsbad, CA). Cells were visualized with a Zeiss Axioplan2 microscope using a CoolSnap HQ charge-coupled-device camera and analyzed using MetaView MetaMorph software.

### GST-Pulldown

Recombinant His-tagged WIP1ΔEx6 (His-WIP1) and phosphatase dead His-tagged D314A-WIP1ΔEx6 (dHis-WIP1) were previously described [64], [66]. GST-Tax was purified as previously described [67]. In brief, cultures were harvested and washed in TBS (10 mM Tris, 150 mM NaCl, pH 7.4) followed by sonication in NETN (100 mM NaCl, 1 mM EDTA, 20 mM Tris-HCl pH 8.0 and 0.5% NP-40) containing 1 mM DTT, 1 mM PMSF, 2 ug/ml each of aprotinin, leupeptin and pepstatin. Insoluble debris was pelleted and the supernatant was incubated with pre-washed Glutathione-Sepharose beads (Amersham, Piscataway, NJ). Beads were washed 3 times in NETN buffer and resuspended in Laemmli buffer for western blot analysis For GST-Tax and His-WIP1 interaction experiments, GST-Tax was bound to prewashed GST-sepharose beads for 1 hr at 4°C in TEE buffer (50 mM Tris-HCl pH 7.9, 1 mM EDTA, 1 mM EGTA). Beads were washed 3 times before adding His-WIP1 and were then incubated for 2 hours rotating at 4°C. Beads were then washed 4 times in TEN100 buffer (20 mM Tris-HCl Ph 7.4, 0.1 mM EDTA, 100 mM NaCl) and resuspended in Laemmli buffer for western blotting.

### In vitro Phosphatase Assay

Phosphopeptides used were synthesized by New England Peptide (Gardner, MA) and are as follows: UNG2 pT31(Ac-AVQGpTGVAGV-Amide), p38-MAPK pT180 (Ac-TDDEMpTGpYVAT-Amide) and H2AX pS139 (Ac-GKKATQApSQEY-Amide). Phosphatase assays were performed as previously described [64]. His-WIP1 (20 ng) or 100 ng dHis-WIP1 was used along with 100 ng of GST or GST-Tax where indicated.

### Quantitative RT-PCR

Cells were treated with UV and allowed to recover for the indicated times prior to harvesting. RNA was purified using an RNeasy kit (Qiagen, Valencia, CA) and 1 ug was used for cDNA synthesis using Superscript III (Invitrogen, Carlsbad, CA) reverse transcriptase according to manufacturer's protocols. One tenth of the cDNA reactions were used for quantitative RT-PCR using SYBR Green PCR Mastermix (Invitrogen, Carlsbad, CA) according to manufacturer's protocol in a Rotor-Gene 3000 machine (Corbett Research, Valencia, CA). Primer sequences for WIP1 amplification were 5′-tagtggtgctcagcctgcaa-3′ and 5′-tctgcgtcgcatggtgagt-3′. GAPDH amplification primers were 5′-ggagtccctgccacactcag-3′ and 5′-ggcccctcccctcttca-3′. WIP1 mRNA was normalized to GAPDH using Rotor-Gene 6 software and relative expression calculated by the standard ddCT method.

### WIP1 Inhibition and Knockdown

CREF-Neo and CREF-Tax cells were transfected with WIP1 siRNA (CGGCAUCGUCGAAGAUAAA) or control non-targeting siRNA (Dharmacon, Chicago,IL) using Oligofectamine (Invitrogen, Carlsbad, CA.) according to manufacturer's directions. Cells were damaged with 30 J/m^2^ UV-C irradiation 48 hours post-transfection and harvested 4 hours later for analysis by western blot. CEM and MT4 cells were treated with 3 µM Compound M (designated #321237 in the diversity set library by the Developmental Therapeutics Program, NCI/NIH) in serum-free media for 1 hour prior to addition of 4-NQO (Sigma, St. Louis, MO) at a final concentration of 2.5 µM.
